# PReS-FINAL-2139: Tapering and withdrawal of tocilizumab in patients with systemic JIA in inactive disease: results from an alternative dosing regimen in the tender study

**DOI:** 10.1186/1546-0096-11-S2-P151

**Published:** 2013-12-05

**Authors:** F De Benedetti, N Ruperto, HI Brunner, A Grom, N Wulffraat, M Henrickson, R Jerath, Y Kimura, AK Kadva, J Wang, A Martini, D Lovell

**Affiliations:** 1 IRCCS Ospedale Ped Bambino Gesú, Rome, Italy; 2 PRINTO, Genoa, Italy; 3 PRCSG, Cincinnati, USA; 4 Genentech, San Francisco, CA, USA; 5 Roche, Welwyn, UK

## Introduction

The TENDER clinical trial is a three-part, 5-year, phase 3 study with tocilizumab (TCZ) in active systemic JIA (sjia). After 2 years of treatments, sjia patients who have maintained clinically inactive disease for 3 months are given the option to participate in an alternative TCZ dosing regimen aimed at spacing the infusions and eventually withdrawing TCZ.

## Objectives

To describe the patients registered to participate in the optional alternative dosing schedule in the TENDER study.

## Methods

To qualify for the optional alternative dosing schedule (OADS), patients had to be in the study for 2 years and have achieved ACR JIA inactive disease status. Among the 112 patients (pts) enrolled, 39 (35%) entered the optional alternative dosing regimen. This entailed a staged prolongation of the time interval between TCZ infusions from 2 weeks (standard interval) to 3 weeks, then 4 weeks, with the option of terminating TCZ after the discontinuation of any treatment.

## Results

23 male and 16 female patients entered the OADS. Their mean baseline characteristics were 14.2 active joints, 15.4 joints with limitation of motion (LOM), physician global VAS score of 58.5, CHAQ-DI score of 1.62 and ESR of 56.8; 15 had fever. Of these 39 patients, 13 patients lost clinically inactive disease status while on the OADS. In these 13 patients, the time to loss of inactive disease status ranged from 1.4 to 16.8 months from initiation of the OADS (n = 4 on 3 weekly dosing, n = 6 on 4 weekly dosing, n = 3 off TCZ). Risk of losing inactive disease status on OADS was similar in patients treated with MTX (6/16, 37.5% flared) and in those not receiving MTX (7/23, 30% flared). Inactive disease status was maintained in 26 of the 39 patients entering the OADS. Present dosing intervals were every 3 weeks in 3 pts and every 4 weeks in 14 pts; 9 pts have been able to discontinue TCZ (range of time since discontinuation: 3.6-13.4 months). At baseline, these 9 pts were clinically similar to other pts entering the OADS (mean characteristics: age 9.1 years, 10.9 active joints, 10.9 joints with LOM, physician global VAS score of 45.4, CHAQ-DI score of 1.42, ESR of 47.2; 4 pts with fever at baseline).

## Conclusion

Patients with sjia who maintain clinically inactive disease status can progressively space TCZ infusions, with one-fourth of them able to discontinue all treatments, including TCZ.

## Disclosure of interest

F. De Benedetti Grant/Research Support from: Abbott, Pfizer, BMS, Roche, Novimmune, Novartis, SOBI, N. Ruperto Grant/Research Support from: Abbott, astrazeneca, BMS, Centocor, Lilly, Francesco Angelini, GSK, Italfarmaco, merckserono, Novartis, Pfizer, Regeneron, Roche, Sanofi Aventis, Schwarz Biosciences, Xoma, Wyeth, Consultant for: Abbott, astrazeneca, BMS, Centocor, Lilly, Francesco Angelini, GSK, Italfarmaco, merckserono, Novartis, Pfizer, Regeneron, Roche, Sanofi Aventis, Schwarz Biosciences, Xoma, Wyeth, Speakers Bureau: Abbott, Boehringer, BMS, Novartis, Astellas, Italfarmaco, medimmune, Pfizer, Roche, H. I. Brunner Consultant for: Novartis, Genentech, medimmune, EMD Serono, AMS, Pfizer, UCB, Janssen, Speakers Bureau: Genentech, A. Grom Grant/Research Support from: Roche, Consultant for: Novartis, N. Wulffraat Grant/Research Support from: Novartis, Roche, Pfizer, abbvie, Consultant for: Novartis, Roche, Pfizer, M. Henrickson: None declared., R. Jerath: None declared., Y. Kimura: None declared., A. K. Kadva Employee of: Genentech, J. Wang Employee of: Roche, A. Martini Grant/Research Support from: Abbott, astrazeneca, BMS, Centocor, Lilly, Francesco Angelini, GSK, Italfarmaco, merckserono, Novartis, Pfizer, Regeneron, Roche, Sanofi Aventis, Schwarz Biosciences, Xoma, Wyeth, Consultant for: Abbott, astrazeneca, BMS, Centocor, Lilly, Francesco Angelini, GSK, Italfarmaco, merckserono, Novartis, Pfizer, Regeneron, Roche, Sanofi Aventis, Schwarz Biosciences, Xoma, Wyeth, Speakers Bureau: Abbott, Boehringer, BMS, Novartis, Astellas, Italfarmaco, medimmune, Pfizer, Roche, D. Lovell Grant/Research Support from: NIH, Consultant for: astrazeneca, Centocor, Janssen, Wyeth, Amgen, Bristol-Meyers Squibb, Abbott, Pfizer, Regeneron, Hoffmann-La Roche, Novartis, Genentech, Speakers Bureau: Roche, Genentech.

